# Mutation of the Ser18 phosphorylation site on the sole *Saccharomyces cerevisiae* UCS protein, She4, can compromise high-temperature survival

**DOI:** 10.1007/s12192-016-0750-0

**Published:** 2016-11-25

**Authors:** Susana Gomez-Escalante, Peter W. Piper, Stefan H. Millson

**Affiliations:** 1 Department of Molecular Biology and Biotechnology, University of Sheffield, Firth Court, Western Bank, Sheffield, S10 2TN, UK; 2 School of Life Sciences, University of Lincoln, Brayford Pool, Lincoln, LN6 7DL, UK

**Keywords:** UCS proteins, She4, UNC45, Hsp90, Temperature stress, Yeast

## Abstract

Folding of the myosin head often requires the joint actions of Hsp90 and a dedicated UNC45, Cro1, She4 (UCS) domain-containing cochaperone protein. Relatively weak sequence conservation exists between the single UCS protein of simple eukaryotes (She4 in budding yeast) and the two UCS proteins of higher organisms (the general cell and smooth muscle UNC45s; UNC45-GC and UNC45-SM respectively). In vertebrates, UNC45-GC facilitates cytoskeletal function whereas the 55% identical UNC45-SM assists in the assembly of the contractile apparatus of cardiac and skeletal muscles. UNC45-SM, unlike UNC45-GC, shares with yeast She4 an IDSL sequence motif known to be a site of in vivo serine phosphorylation in yeast. Investigating this further, we found that both a non-phosphorylatable (S18A) and a phosphomimetic (S18E) mutant form of She4 could rescue the type 1 myosin localisation and endocytosis defects of the yeast *she4Δ* mutant at 39 °C. Nevertheless, at higher temperature (45 °C), only She4 (S18A), not She4(S18E), could substantially rescue the cell lysis defect of *she4Δ* mutant cells. In the yeast two-hybrid system, the non-phosphorylatable S18A and S251A mutant forms of She4 and UNC45-SM still displayed the stress-enhanced in vivo interaction with Hsp90 seen with the wild-type She4 and UNC45-SM. Such high-temperature enforcement to interaction was though lost with the phosphomimetic mutant forms (She4(S18E) and UNC45-SM (S251E)), an indication that phosphorylation might suppress these increases in She4/Hsp90 and UNC45-SM/Hsp90 interaction with stress.

## Introduction

Vertebrates have two forms of cytosolic Hsp90, Hsp90α and Hsp90β. There is evidence that their functions are not completely identical. In many tissues, the stress-induced isoform is Hsp90α whereas Hsp90β is more usually constitutively expressed and seems to be associated with development, long-term cell adaptation and evolution (Sreedhar et al. 2004). In mice, Hsp90β is essential for embryonic development, while the loss of Hsp90α is fully compatible with viability though it causes a block to spermatogenesis (Grad et al. 2011). Zebrafish studies have identified Hsp90α as being highly expressed in striated muscle while Hsp90β predominates in other tissues (Krone et al. 2003). Furthermore, as described below, Hsp90α and Hsp90β also differ in their association with UNC45, Cro1 and She4 (UCS) proteins, cochaperones that cooperate with Hsp90 during the folding of the myosin head.

The importance of UCS proteins was initially apparent from the study of *Caenorhabditis elegans UNC-45* (“UNCoordinated”) mutants. These characteristically display defects in both motility (Barral et al. 1998; Ao and Pilgrim 2000) and cytokinesis during embryogenesis (Kachur et al. 2004). The single *C. elegans* UNC45 protein was subsequently shown to associate with both Hsp90 and myosin (Barral et al. 2002), its action facilitating not just myosin folding but also the regulation of myosin levels by targeting excess or damaged myosin to the proteasome for degradation (Landsverk et al. 2007). *C. elegans* UNC45 has been shown to form linear multimers, a filament assembly scaffold for the direct coupling of myosin folding with myofilament formation and the organisation of sarcomeric repeats (Gazda et al. 2013).

The sole UCS protein of budding yeast, She4, is required for actin cytoskeleton polarisation, endocytosis (Wendland et al. 1996) and the asymmetric messenger RNA (mRNA) localisation of *ASH1* mRNA to daughter cells (Long et al. 1997). She4 interacts with the *Saccharomyces cerevisiae* class I myosins (Myo3 and Myo5) in a temperature-dependent manner (Toi et al. 2003; Wesche et al. 2003). Rng3, the UCS protein of the fission yeast *Schizosaccharomyces pombe*, interacts with both Hsp90 (Mishra et al. 2005) and the class II myosin Myo2 (Lord and Pollard 2004; Mishra et al. 2005), increasing myosin II affinity for actin filaments (Lord et al. 2008). *RNG3* mutants show a 10-fold decrease in Myo5 levels, a 4-fold decrease in cortical actin patches (Lord et al. 2008) and defective cytokinesis (Wong et al. 2000). Thus, while the phenotypes associated with mutations in the *C. elegans* and fungal UCS proteins would seem—at first sight—to differ considerably, all relate to effects on myosin assembly and/or function.

Fungi and worms have just this single UCS protein. However, vertebrates have two—a general cell UNC45-GC (or Unc-45A) expressed in most somatic cells and a striated muscle UNC45-SM (Unc-45B) highly expressed only in the heart and skeletal muscle (Hutagalung et al. 2002; Price et al. 2002). Zebrafish studies have revealed that these UNC45s are not functionally redundant (Comyn and Pilgrim 2012). UNC45-GC plays a role in the cytoskeletal functions of most cells while UNC45-SM has a more specific role in the assembly of the contractile apparatus of cardiac and skeletal muscles (reviewed in Ni and Odunuga 2014; Lee et al. 2014). Furthermore, UNC45-SM associates specifically with Hsp90α, not Hsp90β (Etard et al. 2007). In contrast, UNC45-GC interacts preferentially with Hsp90β in vitro, mediating Hsp90β but not Hsp90α function when chaperoning the progesterone receptor to its hormone-binding state (Chadli et al. 2008).

**Fig. 1:**
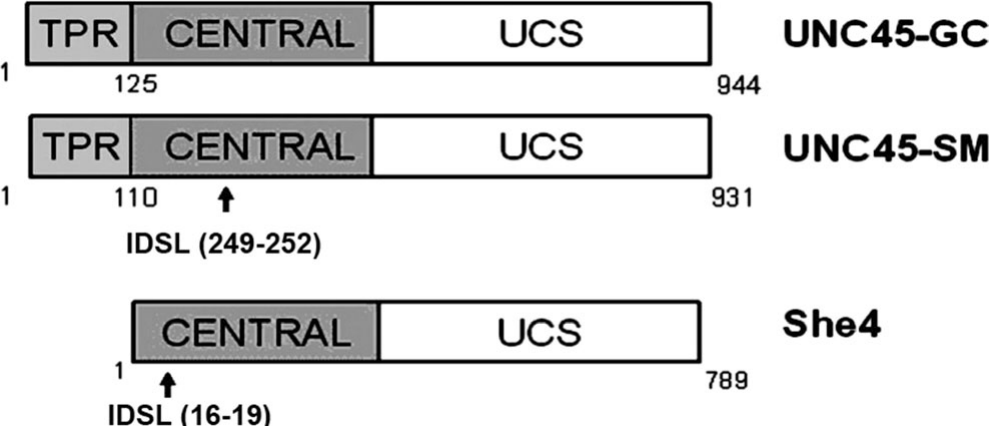
UCS protein domains showing the position of the IDSL motif serine in yeast She4 and human UNC45-SM

UCS proteins are characterised by a C-terminal UCS domain containing several beta-catenin-like repeat sequences (Fig. 1). Indications of how this domain might facilitate the association of the myosin head with its actin filament binding site have emerged from the atomic structure of the yeast She4 dimer (Shi and Blobel 2010). UCS proteins also have a central domain of less defined function and—in the case of the vertebrate and worm proteins—an N-terminal tetratricopeptide (TPR) repeat (Fig. 1). The fungal UCS proteins lack this latter TPR domain, yet they still interact with Hsp90 (Millson et al. 2004; Mishra et al. 2005). Screening for protein phosphorylations in *S. cerevisiae* has revealed that She4 is phosphorylated on Ser18 (Albuquerque et al. 2008). This serine lies within a short IDSL sequence motif that is also to be found in the human UNC45-SM, though not UNC45-GC (Fig. 1). While this motif is not present in many fungal UNC45s, also the *C. elegans* UNC45, it appears relatively conserved in vertebrates (in the zebrafish UNC45-SM it is NDSL(251–255)). We have considered whether this Ser18 phosphorylation might regulate the function of the *S. cerevisiae* She4. Already, there are precedents for the phosphorylation of cochaperones of the Hsp90 system facilitating the “loading” of client proteins onto the chaperone complex. Phosphorylation, then dephosphorylation, of Cdc37 is needed for the efficient presentation of nascent protein kinases to Hsp90 (Mandal et al. 2007; Vaughan et al. 2008). In addition, a Ser361 phosphorylation of Sgt1 inhibits the dimerisation of this Sgt, a dimerisation needed for Sgt1 and Hsp90 to assist the binding of Skp1 to the yeast kinetochore (Bansal et al. 2009). This report describes an investigation into the effects of mutating Ser18 of She4 in vivo in yeast.

## Materials and methods

### Yeast strains and culture

The strains generated for this study are listed in Table 1. DNA cassettes for gene deletion were generated by PCR using hphMX4 (Goldstein and McCusker 1999) as template, and cassettes for C-terminal tagging of genes with green fluorescent protein (GFP) were generated using pUG23 (Niedenthal et al. 1996) as template.

**Table 1.** Yeast strains used in this study

Strain	Genotype	Source
BY4741	MATa *his3Δ1*, *leu2Δ0*, *met15Δ0*, *ura3Δ0*	Euroscarf
BY4741 *she4*∆	BY4741 *she4*Δ::*hphMX4*	This study
BY4741 *SHE4-GFP*	BY4741 *SHE4-GFP*::*HIS3*	This study
BY4741 *MYO5-GFP*	BY4741 *MYO5-GFP*::*HIS3*	This study
BY4741 *MYO5-GFP she4*∆	BY4741 *MYO5-GFP*::*HIS3 she4*Δ::*hphMX4*	This study
BY4741 *MYO5-GFP myo3*∆	BY4741 *MYO5*-*GFP*::*HIS3 myo3*Δ::*hphMX4*	This study

### Engineering yeast for the expression of mutant She4 under native promoter control

*SHE4* sequences were first PCR-amplified from yeast genomic DNA using primers which add a 6xHis-tag at the N-terminus of the gene, then inserted into pCR®-XL-TOPO® (Invitrogen), thus providing the template for the site-directed mutagenesis of Ser18 using the QuickChange Mutagenesis Kit (Stratagene) (primer sequences available on request).

After sequence confirmation, these pCR^®^-XL-TOPO clones containing the genes for either wild-type, S18A or S18E 6xHis-*SHE4* were PCR-amplified using primers *GAAAAGATTACTAAAAAATTAGAATCACGACTAGT*ATGCATCATCATCATCATCATCCACTGTGTGAGAAAGGGA (His-tag sequence underlined) and *TCCATAGAATTCCTGCAGCCCGGGGGATCCACTAGT*TTAGACTTTAATTTTAGCAAG—these having 3′ sequence homologies to the *SHE4*-coding region and 5′ homologies (italicised) to the sequence of pShep next to the *Spe*I restriction site of the latter plasmid. pShep, constructed for this study, is a centromeric *URA3* plasmid designed for expressing genes under the control of *SHE4* promoter and terminator sequences. It comprises pRS416 (Sikorski and Hieter 1989) with inserts of the native *SHE4* promoter (−520 to −1) between its *Not*1 and *Spe*1 sites and the *SHE4* terminator region (+2971 to +3378) between its *Eco*R1 and *Sal*1 sites.

The above PCR-amplified genes for either the wild-type, S18A or S18E 6xHis-She4 were next inserted into yeast by homologous recombination, transforming BY4741 *she4*∆ (Table 1) to uracil prototrophy with the appropriate PCR product and *Spe*I-cleaved pShep. Transformants were initially checked by colony PCR, then protein extracts were analysed by western blotting using an anti-His antibody to confirm similar levels of 6xHis-tagged protein expression.

### Phenotype analysis

Rescue of endocytosis at high temperature was analysed as the uptake of FM4–64, a fluorescent lipophilic styryl dye which stains the vacuole via the endocytic pathway (Smythe and Ayscough 2006). Cells growing on minus uracil dropout medium at 30 °C were incubated with 16 μM FM4–64 (Invitrogen, Ltd.) for 10 min, then chased in the absence of dye for 45 min at 30 or 39 °C. Samples were observed by fluorescence microscopy, acquiring images with the same exposure parameters so as to allow a direct comparison of image intensities.

For heat survival measurements, cultures growing on minus uracil dropout medium at 30 °C were incubated 1 h at 45 °C, survival being measured by plating dilutions on YPD agar plates before and after the 45 °C treatment and counting colonies after 3 days at 30 °C.

### Yeast two-hybrid analysis

*UNC45-SM* was PCR-amplified from its cDNA (image clone 40008187, Geneservice, Cambridge, UK), then inserted into pCR^®^-XL-TOPO^®^ prior to site-directed mutagenesis of Ser251 using the QuickChange Mutagenesis Kit. After sequence confirmation, the full-length wild-type, S251A and S251E *UNC45-SM* genes, as well as the above wild-type, S18A and S18E genes for yeast *SHE4*, were PCR-amplified using primers that add the terminal sequences for homologous recombination with linearised plasmids pADC or pBDC (as detailed in Millson et al. 2005). These PCR products were then transformed, together with the Y2H vectors pADC or pBDC, into the yeast strains used for yeast two-hybrid (Y2H) screening in our earlier studies, PJ69-4a or PJ69-4α respectively (Millson et al. 2003, 2004, 2005). After confirming that the resulting fusions did not self-activate in these PJ69-4 cells (did not appreciably enhance basal expression from the *GAL4* promoter-directed β-galactosidase gene), these transformants were next mated with PJ69-4 cells of the opposite mating type, cells that express either Hsp82-BD, Hsp90α-BD or AD-Myo5. Quantitative analysis of Y2H interaction strength, *GAL4* promoter-directed β-galactosidase gene expression, was as previously described (Millson et al. 2003, 2004, 2005). Control cells were those containing the appropriate pBDC fusion and empty pADC, since basal expression in this system is generally due to the latter plasmid (Millson et al. 2004).

## Results

### Lack of She4 causes defective Myo5 localisation and endocytosis at 39 °C

In this study, the functionality of She4 in yeast was studied as its ability, when expressed in cells of the *she4Δ* mutant, to rescue (i) the defective localisation of the class I myosin Myo5—observed as a functional Myo5-GFP fusion—at 39 °C, (ii) the defective localisation of the fluorescent dye FM4-64 to the vacuolar membrane, also at 39 °C and (iii) cell survival at 45 °C. FM4-64 is a lipophilic styryl dye that is normally rapidly internalised in budding yeast through endocytosis, whereupon it is subsequently targeted to the vacuolar membrane (Smythe and Ayscough 2006).

Myo5, one of the two class I myosins of overlapping function in *S. cerevisiae* (Myo3/5), plays an important role in endocytosis. Whereas no phenotype is apparent with the loss of either these myosins individually, a double *myo3*Δ *myo5*Δ knockout exhibits severe defects in both growth and the organisation of the actin cytoskeleton (Goodson et al. 1996). We confirmed that Myo5 is substantially functional with a C-terminal GFP tag by generating the strain BY4741 *MYO5*-*GFP myo3*Δ (Table 1). This did not display the severe phenotype described for the double *myo3*Δ *myo5*Δ knockout, but instead displayed normal growth and Myo5-GFP localisation to cortical patches at 39 °C. Similarly, She4 is also substantially functional when tagged with GFP, since the BY4741 *SHE4-GFP* strain (Table 1) did not exhibit *she4Δ* mutant phenotypes (unpublished observations).

**Fig. 2:**
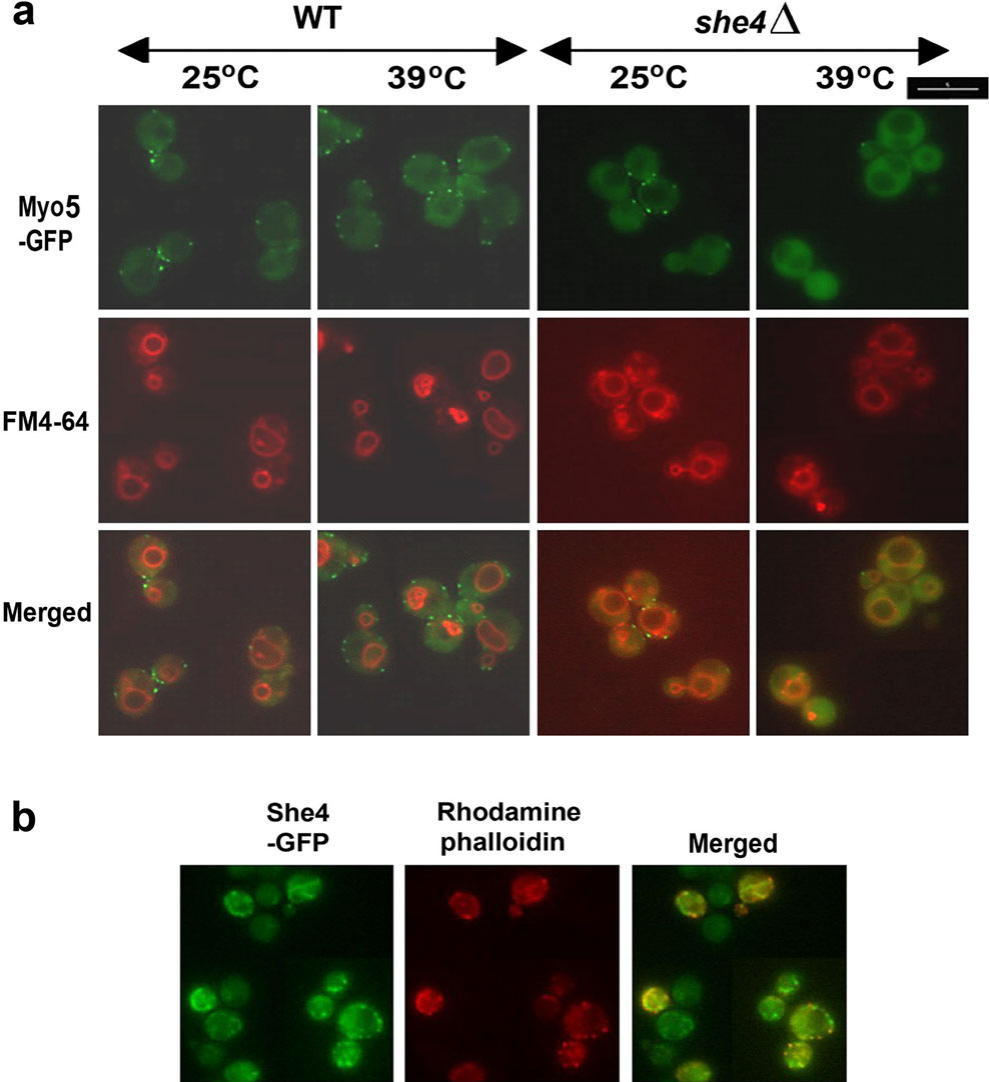
**a** Myo5-GFP localisation and FM4-64 staining in BY4741 *MYO5-GFP* wild-type (WT) or *she4*Δ cells either in growth at room temperature (25 °C) or heat shocked from 25 to 39 °C for 1 h. **b** BY4741 *SHE4-GFP* cells growing at 39 °C were fixed and stained with rhodamine-phalloidin to observe co-localisation with actin. *Scale bar* 5 μm

In yeast, She4 appears especially important for the functioning of class I myosins when cells are subject to stress, since the phenotypes of the *she4Δ* mutant are most apparent at high temperature (Toi et al. 2003; Wesche et al. 2003). We analysed the localisation of Myo5-GFP and FM4-64 in strains BY4741 *MYO5*-*GFP* and BY4741 *MYO5*-*GFP she4Δ* (Table 1), both at 25 °C and in cells subjected to a 39 °C heat shock (Fig. 2a). At the lower temperature, both strains showed Myo5-GFP localisation to cortical patches as observed in earlier studies (Goodson et al. 1996; Toi et al. 2003; Wesche et al. 2003). However, in the cells lacking She4, more of this Myo5-GFP was dispersed through the cytosol, especially at 39 °C when patch-like localisation of the Myo5-GFP was almost completely lost (Fig. 2a). This reflects the action of She4 in facilitating the localisation of Myo5, especially at high temperature. We also found a She4-GFP fusion to be substantially localised to actin cortical patches at 39 °C (Fig. 2b).

Another prominent feature of the *she4Δ* mutant is its defect in endocytosis, apparent from the relatively weak FM4-64 staining of the vacuole at 39 °C in Fig. 2a.

### The in vivo effects of She4(S18A) and She4(S18E) expression

**Fig. 3.**
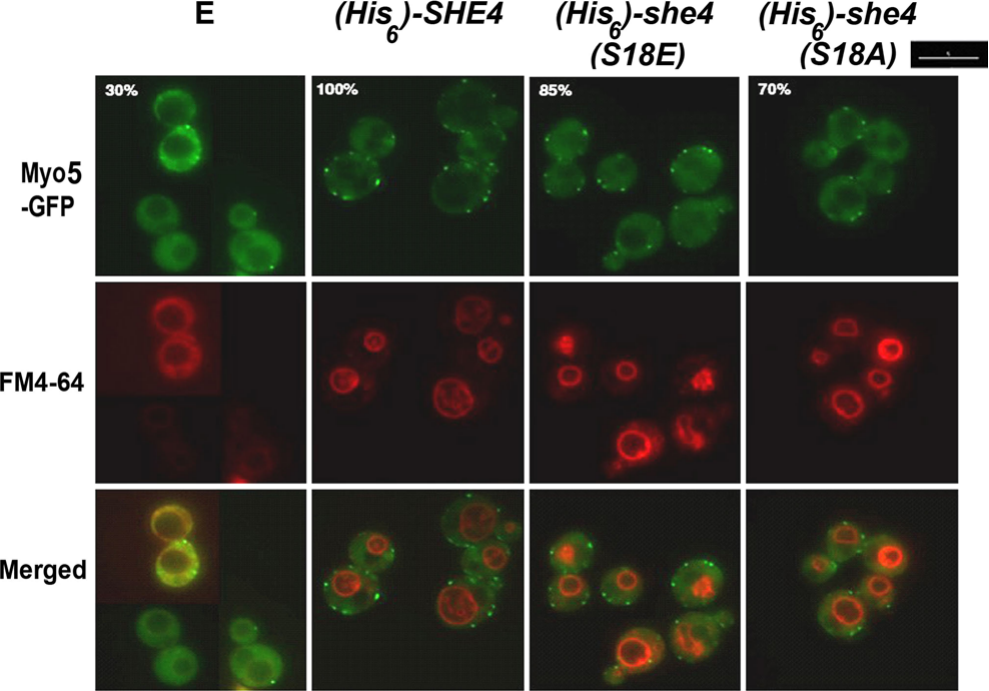
Myo5-GFP localisation and FM4-64 uptake at 39 °C in BY4741 *She4*Δ *MYO5*-*GFP* cells containing either empty pShep vector (E) or pShep-borne genes for 6xHis-She4, 6xHis-She4(S18E) or 6xHis-She4(S18A). The percentages relate to the total number of cells that displayed cortical patches. *Scale bar* 5 μm

Genes for wild-type as well as S18A and S18E mutant forms of 6xHis-She4 were inserted into strain BY4741 *MYO5*-*GFP she4Δ* (Table 1), these genes being under the control of the native *SHE4* gene promoter and terminator sequences and carried on a centromeric plasmid vector (see Materials and methods). Both Myo5-GFP and FM4-64 staining in the resultant *she4Δ* cells containing either empty pShep vector, the non-mutant 6xHis-She4 or S18A and S18E mutant forms of 6xHis-She4 was then analysed (Fig. 3).

All three (wild type, S18E and S18A) forms of 6xHis-She4 were observed to rescue Myo5-GFP movement from the cytosol to organised patches in *she4Δ* cells at 39 °C (Fig. 3). Analysing FM4-64, all three forms of 6xHis-She4 were also found to have facilitated the targetting of the FM4-64 endocytic marker to the vacuole (Fig. 3). Therefore, both 6xHis-She4(S18E) and 6xHis-She4(S18A) were able to provide substantial rescue of the defective endocytosis in these *she4Δ* cells at 39 °C (Fig. 2a).

**Fig. 4.**
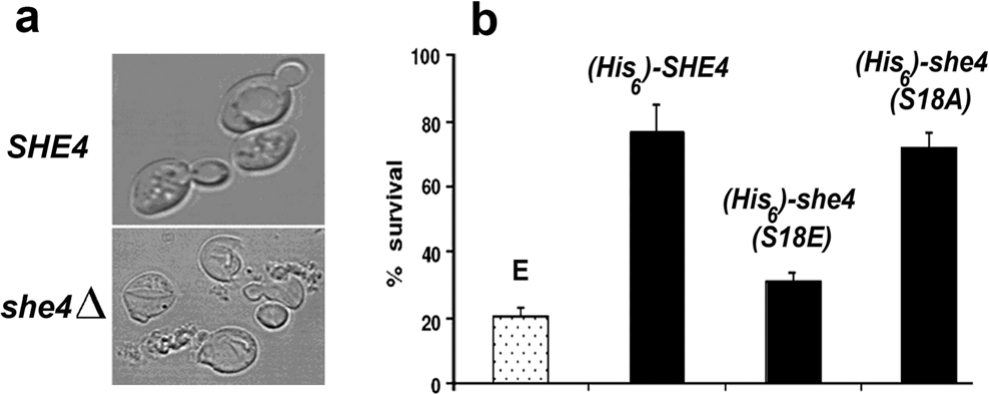
**a** Phase contrast images of BY4741 WT and *she4*Δ after a 1-h incubation at 42 °C. **b** Percentage of cells containing empty vector (E) or expressing 6xHis-She4, 6xHis-She4(S18E) or 6xHis-She4(S18A), which survived a 1-h heat stress at 45 °C (mean and SD from three separate experiments)

One can be reasonably confident that the above analysis relates to viable cells since 39 °C—the maximum growth temperature of BY4741 cells—represents a supraoptimal, yet sublethal heat stress, conditions under which the *she4Δ* mutant shows no enhanced loss of viability. Also, the cells observed here were only subjected to this temperature for less than 2 h during the microscopy. We noted, however, that the *she4Δ* mutant exhibits a strong phenotype of cell lysis at more stressful temperatures (Fig. 4a), losing viability much more rapidly than the wild type. Expressions of both the wild type and the non-phosphorylatable S18A mutant form of 6xHis-She4 were found to restore viability to *she4Δ* cells maintained at 45 °C, whereas the expression of the phosphomimetic mutant 6xHis-She4(S18E) provided little rescue of viability under the same conditions (Fig. 4b). This is an indication that the S18 phosphorylation of She4 may act to modulate the operation of this myosin-specific cochaperone (see Discussion).

### Yeast two hybrid analysis

**Fig. 5.**
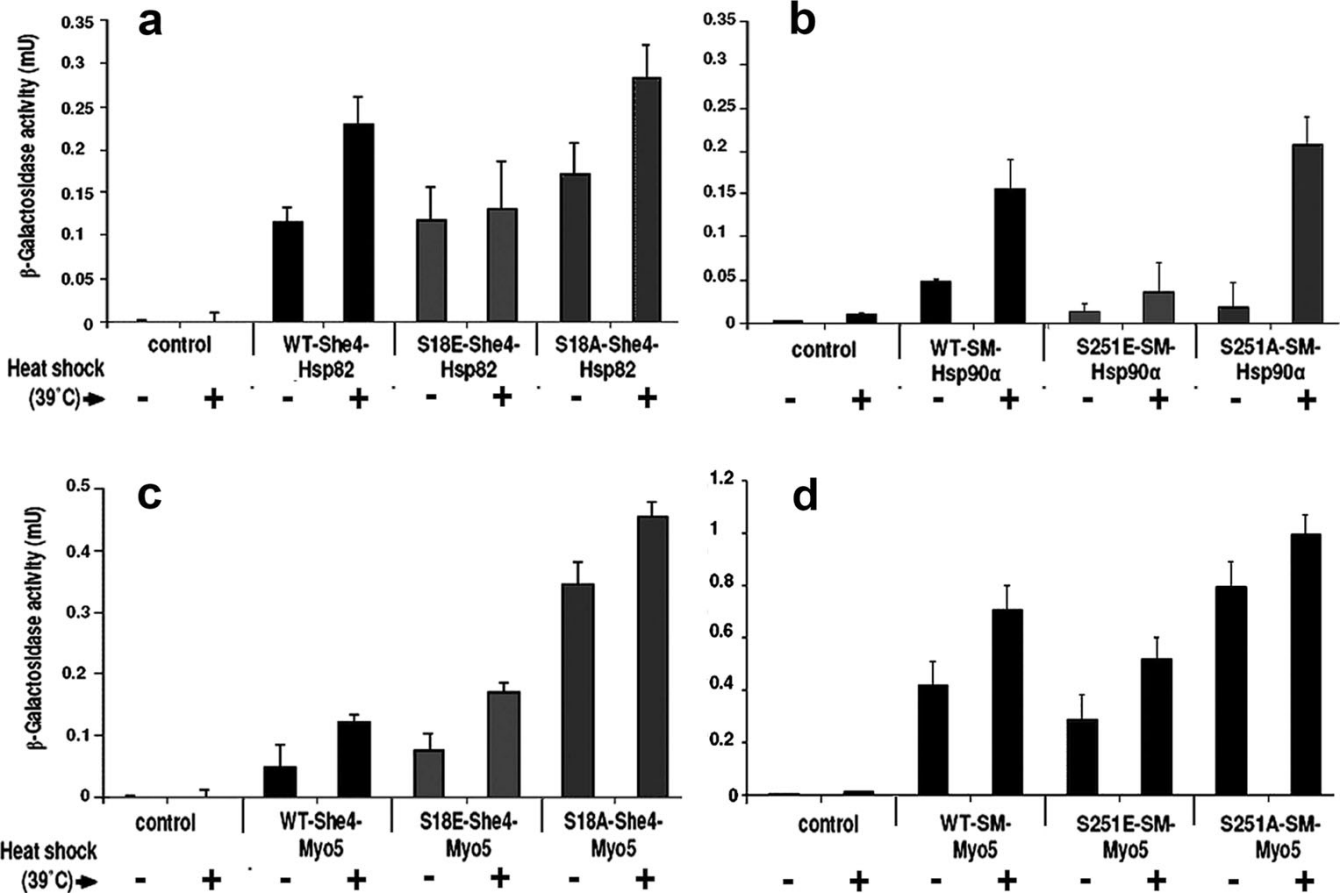
Y2H interactions measured at 25 °C (−) and 39 °C (+). **a** Hsp82-BD with She4 WT, S18A and S18E. **b** Hsp90α-BD with WT, S251E and S251A mutant forms of human AD-UNC45-SM. **c** AD-Myo5 with She4-BD WT, S18A and S18E. **d** AD-Myo5 with WT, S251E and S251A mutant forms of human BD-UNC45-SM. Data shown are the mean and SD of eight replicate assays from the same cell culture

The effects of stress on the in vivo interactions of She4 can be analysed using the yeast two-hybrid (Y2H) system. In this system, the Gal4 activator domain (AD)-She4 fusion displays a temperature-reinforced interaction in vivo with the Hsp82-Gal4 DNA-binding domain (BD) fusion (Millson et al. 2004), the latter a functional form of Hsp90 chaperone (Millson et al. 2003). We investigated whether the S18A and S18E mutant forms of AD-She4 would still display this interaction. As shown in Fig. 5a, the non-phosphorylatable She4(S18A) showed an increased Hsp90 interaction with heat shock in this Y2H system that was comparable to that displayed by the wild-type She4. In contrast, the phosphomimetic mutant She4(S18E) showed no appreciable increase in its Y2H interaction with Hsp90 at 39 °C (Fig. 5a). This is an indication that the Ser18 phosphorylation of She4 might be acting to suppress any reinforcement of She4/Hsp90 binding upon stress.

In the light of this result, we also analysed in the Y2H system the corresponding wild-type, S251A and S251E mutant forms of the human UNC45-SM, studying their interaction with the human Hsp90α (in zebrafish, UNC45-SM appears to associates specifically with the Hsp90α isoform of Hsp90; Etard et al. 2007). As with the corresponding native yeast proteins, Y2H interaction of the human Hsp90α with the wild-type and non-phosphorylatable S251A mutant UNC45-SM was markedly enhanced when the Y2H strains were exposed to heat stress (Fig. 5b). However, the phosphomimetic S251E mutant form of UNC45-SM largely lacked any stress reinforcement of this interaction (Fig. 5b), a result even more marked than that obtained with the corresponding phosphomimetic (S18E) mutant form of She4 (Fig. 5a).

We also analysed how these mutations in She4 and UNC45-SM affect the Y2H interaction with yeast type 1 myosin, Myo5 (Fig. 5c, d). These interactions, modestly enhanced by stress, appeared stronger with the S18A and S251A mutant forms of She4 and UNC45-SM than with the corresponding phosphomimetic S18E and S251E mutant forms of these two UCS proteins (Fig. 5c, d).

## Discussion

Yeast She4 is known to be phosphorylated on Ser18 in vivo (Albuquerque et al. 2008). This serine lies within a short sequence motif (IDSL; Fig. 1) which is also present in the human UNC45-SM, despite the limited sequence conservation between these UCS proteins of yeast and man. This report describes an investigation into the in vivo effects in yeast of mutating this Ser18 residue of She4 to either a non-phosphorylatable alanine or a phosphomimetic glutamic acid, as well as the effects on in vivo interactions in the Y2H system of similarly mutating this Ser18 in yeast She4 and the corresponding Ser251 in the IDSL motif of the human UNC45-SM.

Fluorescence microscopic analysis of Myo5-GFP and FM4-64 revealed that the S18E and S18A mutant forms of 6xHis-She4 were still able to provide substantial rescue of the type 1 myosin localization and endocytosis defects apparent in *she4Δ* cells at 39 °C (Fig. 3). It was at a higher, more stressful temperature—conditions where *she4Δ* cells normally lyse—that our study uncovered the strongest phenotypic difference for the cells expressing the wild-type and (S18A) mutant 6xHis-She4 as compared to those expressing 6xHis-She4(S18E). Only 6xHis-She4(S18A) could provide the rescue of *she4Δ* cell viability at high temperature seen with the expression of the wild-type 6xHis-She4 (Fig. 4b). This may relate to the Y2H analysis, where it was evident that She4(S18E) had lost the capacity to engage in a temperature-enhanced interaction in vivo with Hsp90, a reinforcement that was still displayed by the non-phosphorylatable She4(S18A) (Fig. 5a). The Y2H interactions of the corresponding human proteins, UNC45-SM/Hsp90α and UNC45-SM(251A)/Hsp90α, show even greater reinforcement as the Y2H strains are exposed to high temperature, but again this was lost with the corresponding phosphomimic mutant form of UNC45-SM (UNC45-SM(251E)/Hsp90α; Fig. 5b).

No evidence has yet emerged to suggest that protein phosphorylation plays a role in UCS protein action. Only at 45 °C were we able to identify a strong phenotypic difference between the cells expressing 6xHis-She4(S18E) as compared to those with 6xHis-She4(S18A) (Fig. 4b). This S18E mutation also compromises the stress reinforcement to the interaction with Hsp90 in the Y2H system (Fig. 5a). In conclusion, our study indicates that Ser18 phosphorylation of She4 is non-essential for She4 to facilitate Myo5 localisation and endocytosis at 39 °C (Fig. 4a), yet also reveals that the S18E mutant form of She4 is not devoid of phenotype. The latter finding suggests that the phosphorylation/dephosphorylation of Ser18 might be involved in the fine tuning of She4 action and become especially important at extreme temperatures, conditions under which She4 is essential for cell viability (Fig. 4).

## Abbreviations

UCS protein: A protein that contains the domain shared by UNC45, Cro1 and She4
TPR: Tetratricopeptide
GFP: Green fluorescent protein
BD: Gal4 DNA-binding domain
AD: Gal4 activator domain
Y2H: Yeast two-hybrid
